# Vancomycin Flushing Syndrome Due to Oral Vancomycin in Chronic Kidney Disease Patients: A Case Report

**DOI:** 10.7759/cureus.28024

**Published:** 2022-08-15

**Authors:** Swarada Yadav, Muhammad Usman Hashmi, Sundeep Shah, Abeer Sarwar, Ramy Ibrahim

**Affiliations:** 1 Internal Medicine, University of Alabama at Birmingham, Birmingham, USA; 2 Internal Medicine, Nishtar Medical University Hospital, Multan, PAK; 3 Internal Medicine/Nephrology, Premier Medical Associates, The Villages, USA; 4 Internal Medicine, Fatima Memorial College of Medicine and Dentistry, Lahore, PAK; 5 Research, Premier Medical Associates, The Villages, USA

**Keywords:** infectious colitis, chronic kidney disease (ckd), clostridium difficle infection, adr- adverse drug reactions, oral vancomycin

## Abstract

Vancomycin flushing syndrome is a known complication of vancomycin. It is commonly seen with intravenous (IV) use but is also documented with oral use. We aim to describe a case of a chronic kidney disease (CKD) patient who developed vancomycin flushing syndrome secondary to oral vancomycin use.

Our case is about an immunosuppressed 68-year-old man who received oral vancomycin for pseudomembranous colitis, which was caused by *Clostridium difficile*. On the eighth day of the treatment, the patient experienced pruritus and an erythematous rash, which was diagnosed as vancomycin flushing syndrome, and the oral vancomycin was immediately discontinued. Upon the discontinuation of the drug, the rash disappeared, thus confirming the diagnosis. The patient’s status of chronic kidney disease stage four resulted in reduced clearance of the drug, thus causing the adverse effect.

This case highlights the importance of prophylaxis to prevent vancomycin flushing syndrome in a chronic kidney disease patient. Vancomycin flushing syndrome is commonly seen after the initiation of vancomycin, ciprofloxacin, or amphotericin B. Prompt diagnosis and management are required to prevent the complications due to this condition.

## Introduction

Vancomycin is commonly used for infections arising from methicillin-resistant *Staphylococcus aureus* (MRSA), Corynebacterium species, *Clostridium difficile*, and resistant strains of *Streptococcus pneumoniae* [1]. Vancomycin flushing syndrome is commonly associated with the rapid infusion of intravenous (IV) vancomycin [2]. This syndrome involves pruritus, erythematous rash, wheezing, flushing, and hypotension [1,2]. This phenomenon is also caused by other drugs like ciprofloxacin and amphotericin B [2,3]. The reaction may be seen either during the first exposure or after multiple exposures [3]. Vancomycin flushing syndrome is commonly known to be caused by intravenous infusion but rarely clinical cases have been reported due to oral or local vancomycin use [1,2,4]. When mild, vancomycin flushing syndrome is easy to treat. Once diagnosed, vancomycin should be stopped immediately and diphenhydramine hydrochloride should be administered to stop further advancement of the symptoms [1,3,4]. If more severe symptoms like hypotension are seen, it may need IV fluids and vasopressors [1]. The available medical literature on oral vancomycin and the development of vancomycin flushing syndrome is scarce. Therefore, the purpose of reporting this case is to raise awareness among fellow healthcare professionals. Here, we describe a case of vancomycin flushing syndrome due to the use of oral vancomycin.

## Case presentation

**Figure 1:**
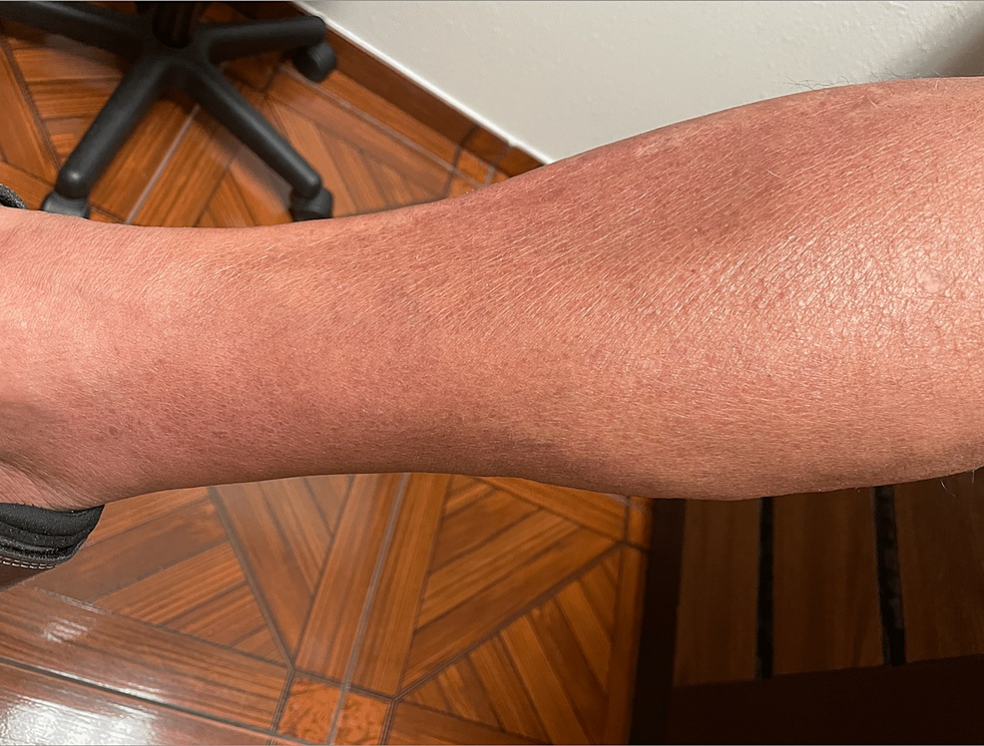
Photograph of the patient's leg which shows erythematous rash caused due to vancomycin flushing syndrome

An immunosuppressed 68-year-old male with a known diagnosis of chronic kidney disease (CKD) due to Wegener's granulomatosis disease was in the hospital due to chronic diarrhea and received metronidazole for the same. The patient is taking furosemide 80 milligrams (mg) once a day and metoprolol extended release 25 mg once a day as long-term medication. One week after the initiation of therapy, the patient developed moderate lower abdominal pain. Diarrhea did not show any improvement. The patient also admitted a change in bowel consistency and frequency. The patient reported a frequency of seven to eight watery bowel movements per day. The stool was mixed with mucus, and there was no blood in the stool. The rest of the review of the system, including cardiovascular, respiratory, and renal, was unremarkable except for the estimated glomerular filtration rate (eGFR) being 23 and serum creatinine being 3.4 mg/dL. His history, recent hospital admission, and concurrent antibiotic use suggested a provisional diagnosis of pseudomembranous colitis. To confirm the diagnosis, a stool exam was performed to detect toxin A and B antigens. Upon the confirmation of *C. difficile* with a positive antigen test, the patient received vancomycin 125 mg per oral six-hourly with a plan to continue it for 10 days. Initially, the patient responded to antibiotic therapy. However, on the eighth day of the antibiotic therapy, he developed generalized pruritus, an erythematous, non-blanching rash on the face, neck, and upper extremities, as demonstrated in Figure 1. The clinical presentation in context with the use of oral vancomycin suggested the vancomycin flushing syndrome.

**Figure 2:**
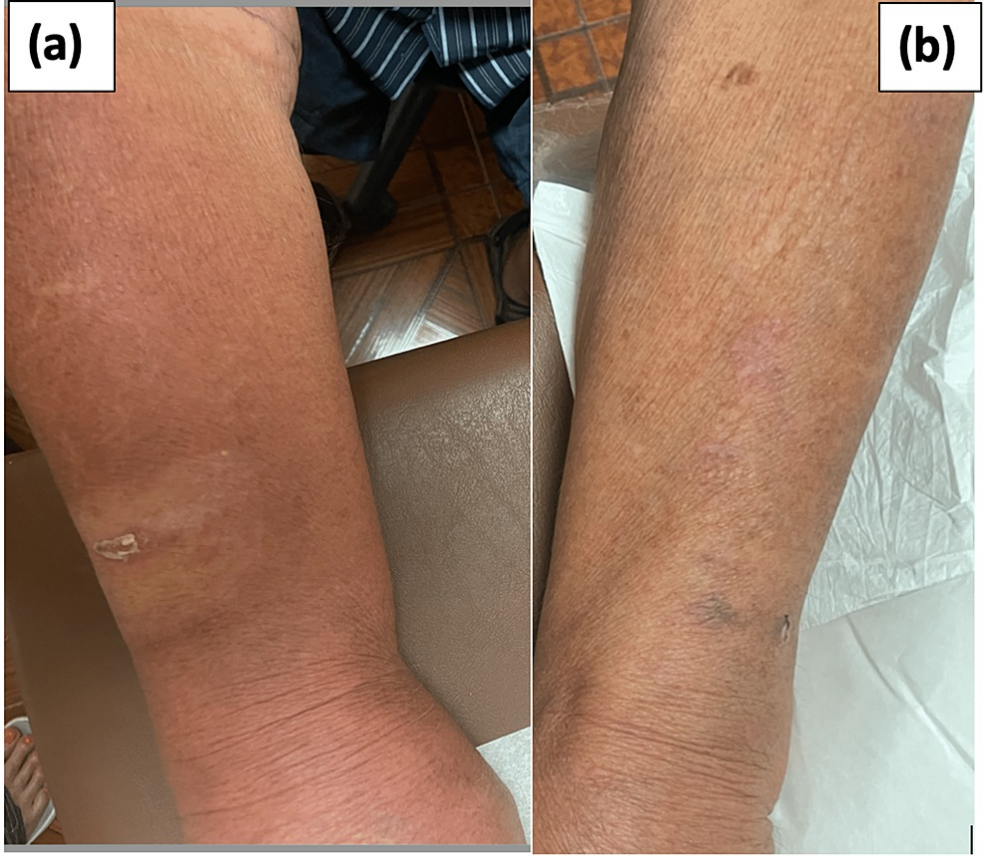
Photographs show the patient's hand: (a) erythematous rash due to vancomycin flushing syndrome; (b) after discontinuation of oral vancomycin

Consequently, the patient received conservative management in the form of antihistamine drugs. The offending agent, oral vancomycin, was stopped. The team also performed continuous noninvasive hemodynamic and pulse oximetry monitoring for the early detection of any severe complications. The rash resolved over the course of the next few days, as shown in Figure 2, which confirmed the diagnosis of vancomycin flushing syndrome.

A few days later, the patient started having diarrhea again. With the multiple days of recent antibiotic therapy and immunocompromised status, candidal colitis was suspected as a likely diagnosis. The patient received fluconazole for the next 14 days, and his symptoms improved significantly after the commencement of antifungal therapy. This provided evidence that the use of broad-spectrum antibiotics caused an overgrowth of selective skin flora and subsequent intestinal colonization led to the development of candidal colitis.

## Discussion

Vancomycin flushing syndrome is a life-threatening but known adverse drug effect with significant complications [1,3]. It is caused by vancomycin-induced activation of mast cells, which release histamine independently of antibodies [2]. It is commonly seen with the use of intravenous vancomycin. However, it has also been documented with oral use [2]. In patients with kidney dysfunction, oral vancomycin use leads to a detectable serum concentration of the drug [5,6]. Vancomycin is not absorbed easily through the gastrointestinal tract, which makes it an ideal drug of choice for pseudomembranous colitis. However, a few cases are documented that have shown toxic vancomycin levels following oral administration [6,7]. Vancomycin is eliminated renally, and about 80% of the dose is cleared unchanged via urine [5,8]. In a patient with existing kidney disease, there is an increased likelihood of developing adverse effects from vancomycin [5,6]. Thus, it is important to take prophylactic measures to prevent vancomycin flushing syndrome, like an oral antihistamine [6]. Oral antihistamines are known to reduce the adverse effects of vancomycin by blocking mast cell activation [6,8]. The management of red syndrome depends on the severity of the symptoms. The mild symptoms can be closely monitored. Oral antihistamines are the drug of choice for moderate symptoms. The severe symptoms need admission and treatment with intravenous fluids, epinephrine, diphenhydramine, famotidine, and symptomatic medications along with continuous hemodynamic monitoring during infusion [7].

The patient in the discussed case has existing chronic kidney disease with an eGFR of 23. The patient was diagnosed with a *C. difficile* infection with a positive stool toxin A and B antigen. He was then started on oral vancomycin 125 mg with the plan of continuing it for 10 days. On the eighth day of the treatment, the patient developed an erythematous and pruritic rash, which was diagnosed as vancomycin flushing syndrome. The drug was immediately stopped and, as the symptoms were mild, no additional treatment was initiated. The rash reduced and almost disappeared upon stopping the medication, which confirmed the diagnosis. There have been case reports which have shown that reduced renal clearance in patients who are given oral vancomycin treatment may play a part in causing vancomycin flushing syndrome[8]. Studies have shown varied vancomycin excretion rates with alterations in renal function, thus making it difficult for dose titration in such patients [6]. Thus, it is important to couple vancomycin with prophylactic antihistamines to prevent vancomycin flushing syndrome, especially in kidney disease patients.

## Conclusions

There has been an increase in cases of pseudomembranous colitis, leading to the frequent use of vancomycin to treat it. The use of oral vancomycin is associated with the development of vancomycin flushing syndrome. Although it is a rare occurrence, it can result in potentially life-threatening complications. It can be diagnosed clinically, and discontinuation of the drug shows immediate improvement. From our literature review, we have concluded that patients with impaired renal function are more prone to developing vancomycin flushing syndrome. This case exhibits the need for close observation of the adverse reaction of vancomycin as it could be life-threatening. It may lead to rapid cardiovascular deterioration of the patient. A thorough clinical assessment with laboratory investigations is necessary for the diagnosis and treatment of the condition. Timely diagnosis and management, which includes discontinuation of vancomycin and other symptomatic treatments, is necessary to prevent complications.
