# Correction: Vancomycin Flushing Syndrome Due to Oral Vancomycin in Chronic Kidney Disease Patients: A Case Report

**DOI:** 10.7759/cureus.c79

**Published:** 2022-10-20

**Authors:** Swarada Yadav, Muhammad Usman Hashmi, Sundeep Shah, Abeer Sarwar, Ramy Ibrahim

**Affiliations:** 1 Internal Medicine, University of Alabama at Birmingham, Birmingham, USA; 2 Internal Medicine, Nishtar Medical University Hospital, Multan, PAK; 3 Internal Medicine/Nephrology, Premier Medical Associates, The Villages, USA; 4 Internal Medicine, Fatima Memorial College of Medicine and Dentistry, Lahore, PAK; 5 Research, Premier Medical Associates, The Villages, USA

This article has been corrected with the consent of the authors to replace the term "red man syndrome" with "vancomycin flushing syndrome". In recent years, the term "red man syndrome" has fallen out of favor as it is now widely considered to be racist (and also outdated, and just plain unhelpful). The editorial office was alerted to this issue by a concerned reader, who we graciously thank for the insight. The authors are in full agreement regarding this change and together with the editorial office regret any harm caused by the use of the word.

